# Supplementary material for: Argyrodite-type advanced lithium conductors and transport mechanisms beyond paddle-wheel effect
Source: Nat Commun. 2022 Apr 19;13:2078. doi: 10.1038/s41467-022-29769-5 (PMC9019101; doi:10.1038/s41467-022-29769-5)
Supplement: Supplementary file 1 — Supplementary Information [file 41467_2022_29769_MOESM1_ESM.pdf]

Supplementary Information

Argyrodite-Type Advanced Lithium Conductors and Transport Mechanisms beyond Peddle-wheel Effect

H. Fang *et al*.

Supplementary Figures 1-12 that help the discussion in the main paper.

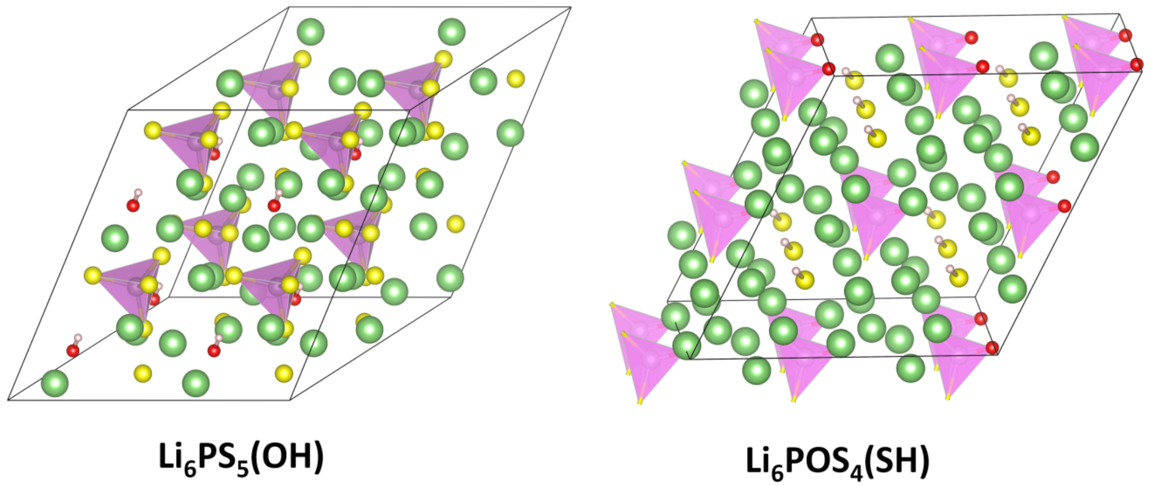

Supplementary Figure 1 Optimized structures of $Li_6PS_5(OH)$ containing OH^- (rod-alike: O in red and H in pink) and PS_4^{3-} (tetrahedron: P in dark red and S in yellow) clusters compared to its ground-state structure $Li_6POS_4(SH)$ containing rod-alike SH- clusters and distorted tetrahedra of POS_3^{3-} (in red). The $Li_6PS_5(OH)$ configuration is 140 meV/formula higher in energy.

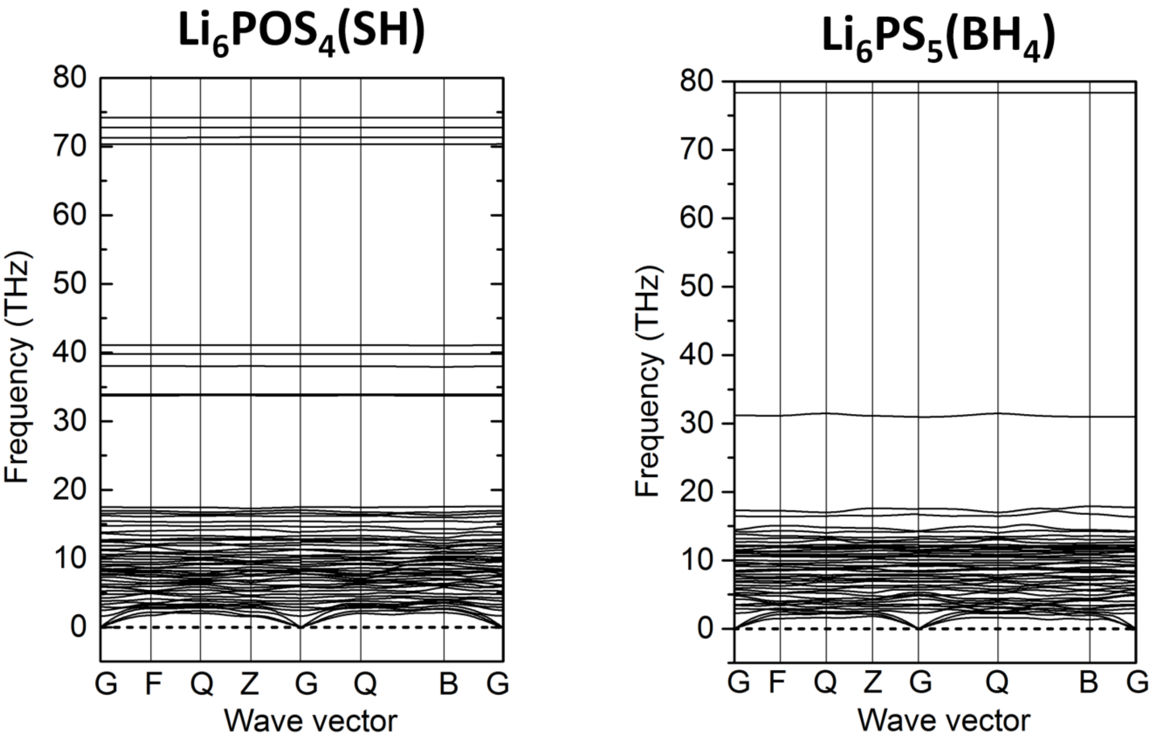

Supplementary Figure 2 Calculated phonon dispersion relations for the triclinic phases of $Li_6POS_4(SH)$ and $Li_6PS_5(BH_4)$, suggesting they are lattice dynamically stable. The wave vectors are: G (0.0,0.0,0.0), F (0.0,0.5,0.0), Q (0.0,0.5,0.5), Z (0.0,0.0,0.5) and B (0.5,0.0,0.0).

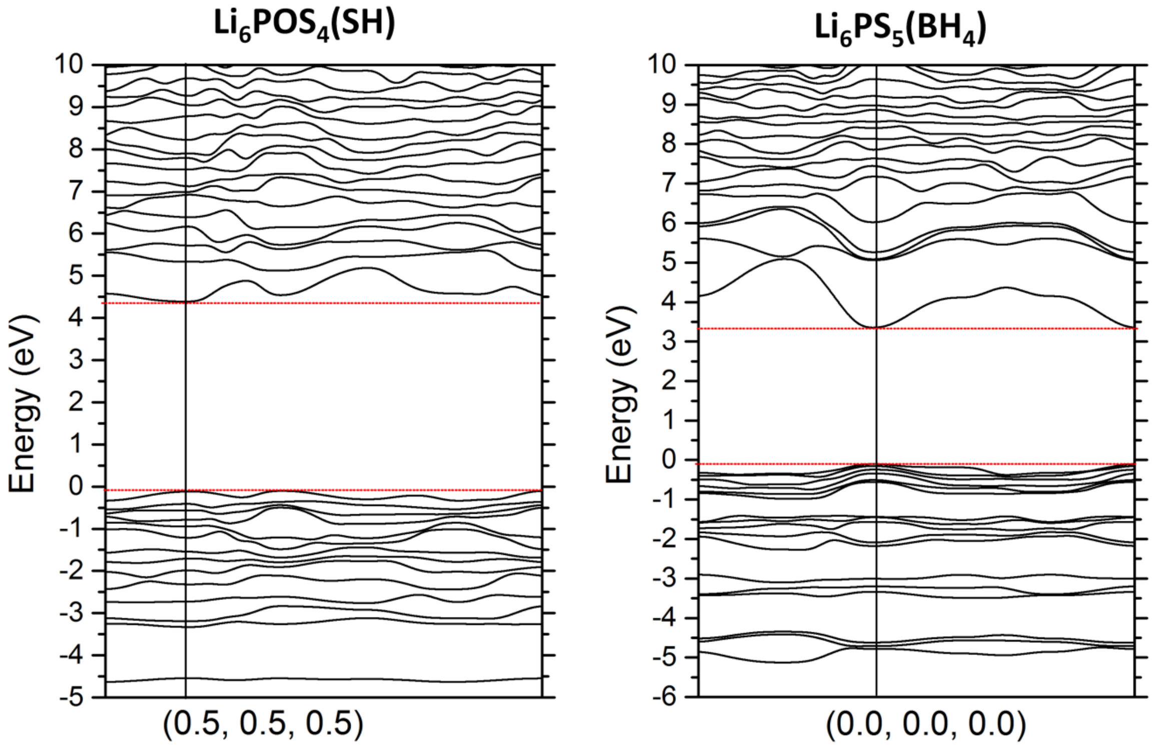

Supplementary Figure 3 Calculated electronic structures for $Li_6POS_4(SH)$ and $Li_6PS_5(BH_4)$ by HSE06 functional. The red lines show the band gap in each case.

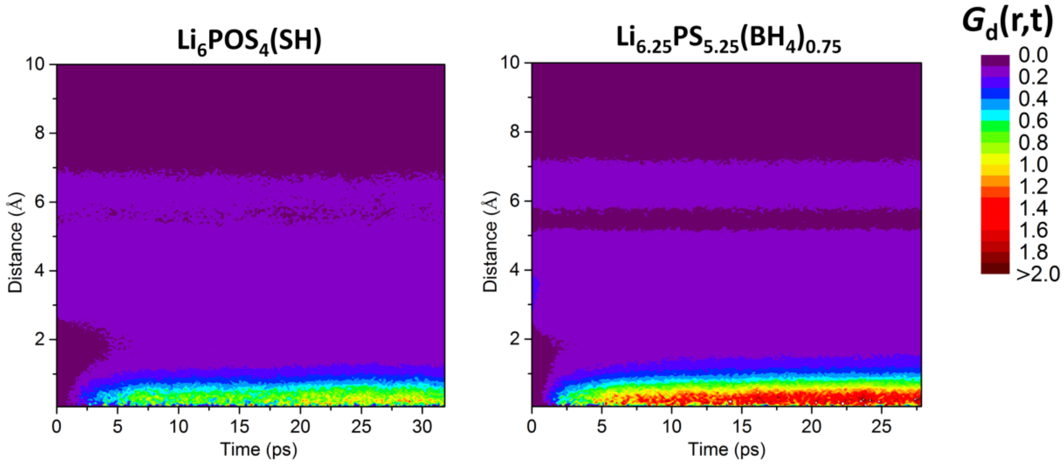

Supplementary Figure 4 (**A**) Calculated van Hove time correlation functions $G_d(\mathbf{r},t)$ (see Method in the paper) for the studied lithium conductors. Compared to $Li_{10}GeP_2S_{12}$ (LGPS), $Li_7La_3Zr_2O_{12}$ (LLZO) and $Li_{1.3}Al_{0.3}Ti_{1.7}(PO_4)_3$ (LATP) with strong collective modes of Li-ions [20], there is only significant correlation between the nearest neighbor lithium in $Li_6POS_4(SH)$ and $Li_{6.25}PS_{5.25}(BH_4)_{0.75}$.

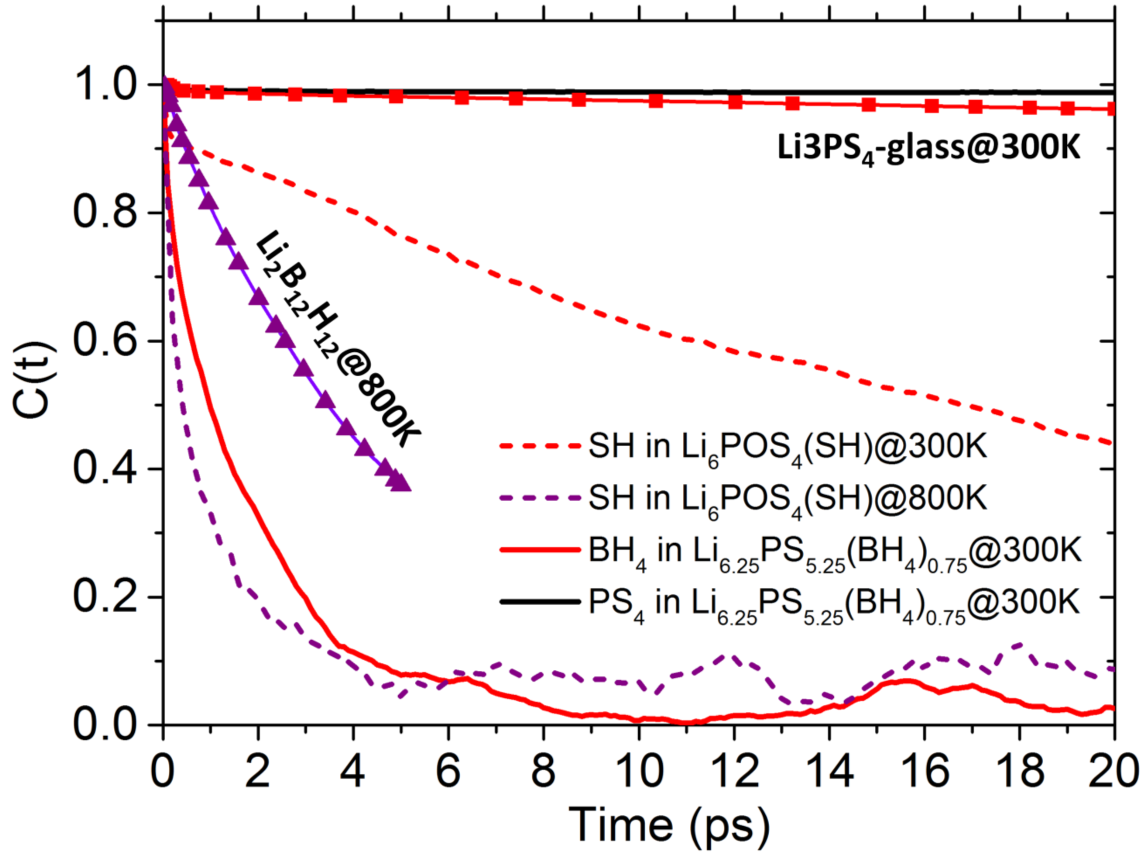

Supplementary Figure 5 Calculated angular time-correlation functions $C(t) = \langle \boldsymbol{u}(t) \bullet \boldsymbol{u}(t + t') \rangle$ for SH^- and BH_4^- in $Li_6POS_4(SH)$ and $Li_{6.25}PS_{5.25}(BH_4)_{0.75}$, respective, compared to those of PS_4^{3-} and $B_{12}H_{12}^{2-}$ in Li_3PS_4 glass [Smith, J. G.; Siegel, D. J. Low-temperature paddlewheel effect in glassy solid electrolytes. Nature Communications 11, 1483, 2020] and $Li_2B_{12}H_{12}$ [19], respectively. $\boldsymbol{u}(t)$ is the unit vector from the center of mass of a cluster to its vertices at time *t*, *e.g.*, the unit vector from S to H in SH and from B to H in BH_4.

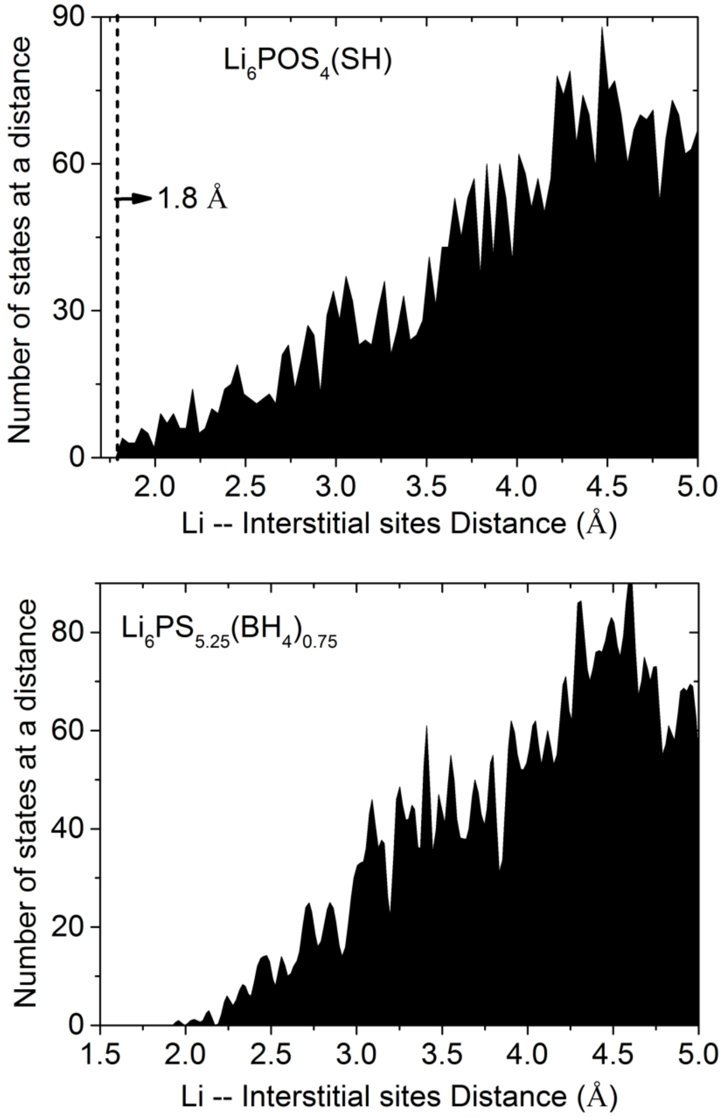

Supplementary Figure 6 Number of Li--Interstitial pairs vs. the Li--Interstitial distance, with the Li-ion and the interstitial site located separately in the neighboring sulfur blocks, corresponding to possible inter-diffusions. The interstitial site counted here must have enough space to accommodate at least the size of one Li-ion according to its Shannon-type ionic radius (in crystal) of 0.9 Å. These are obtained by conducting a topology analysis on the structure snapshots from molecular dynamics simulations lasting 100 ps. The minimal distance in each case is adopted as the distance threshold for picking out the long-lived long-distance events.

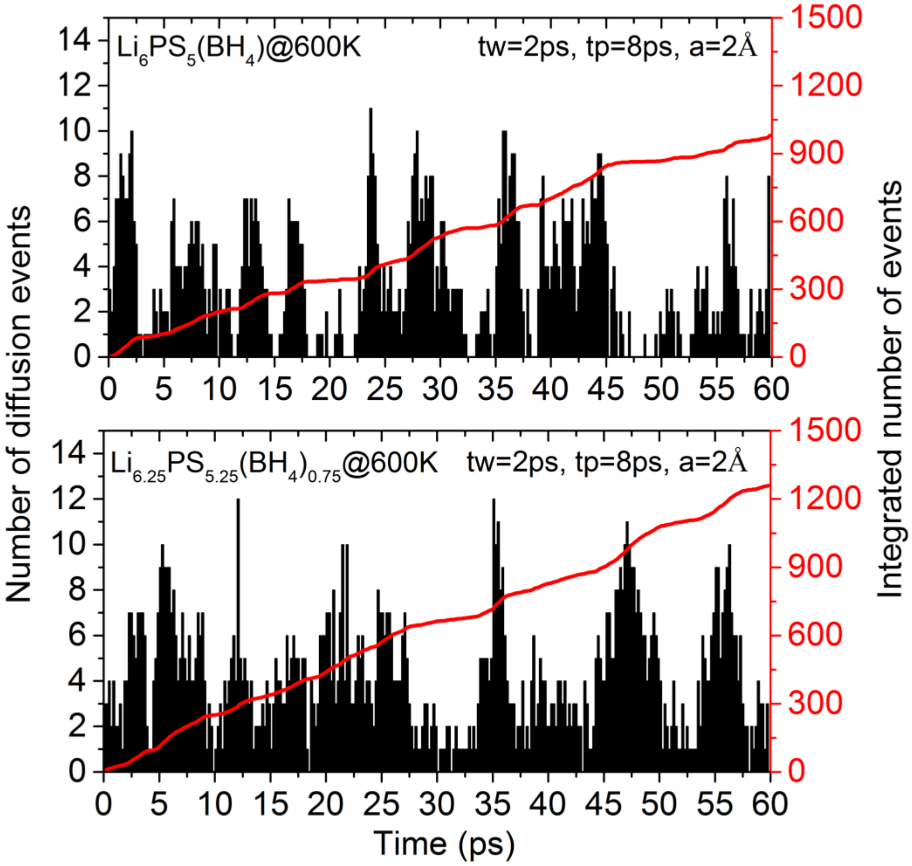

Supplementary Figure 7 Identified number of long-lived long-distance diffusion events in $Li_6SP_5(BH_4)$ at simulation temperature of 600 K compared to that in the non-stoichiometric $Li_{6.25}SP_{5.25}(BH_4)_{0.75}$. It is clear that the latter has many more diffusion events than the former, explaining the much higher ionic conductivity of the latter. The integrated number of the diffusion events (red line in each case) over 60 ps is 979 in $Li_6SP_5(BH_4)$, while it is 1259 in $Li_{6.25}SP_{5.25}(BH_4)_{0.75}$.

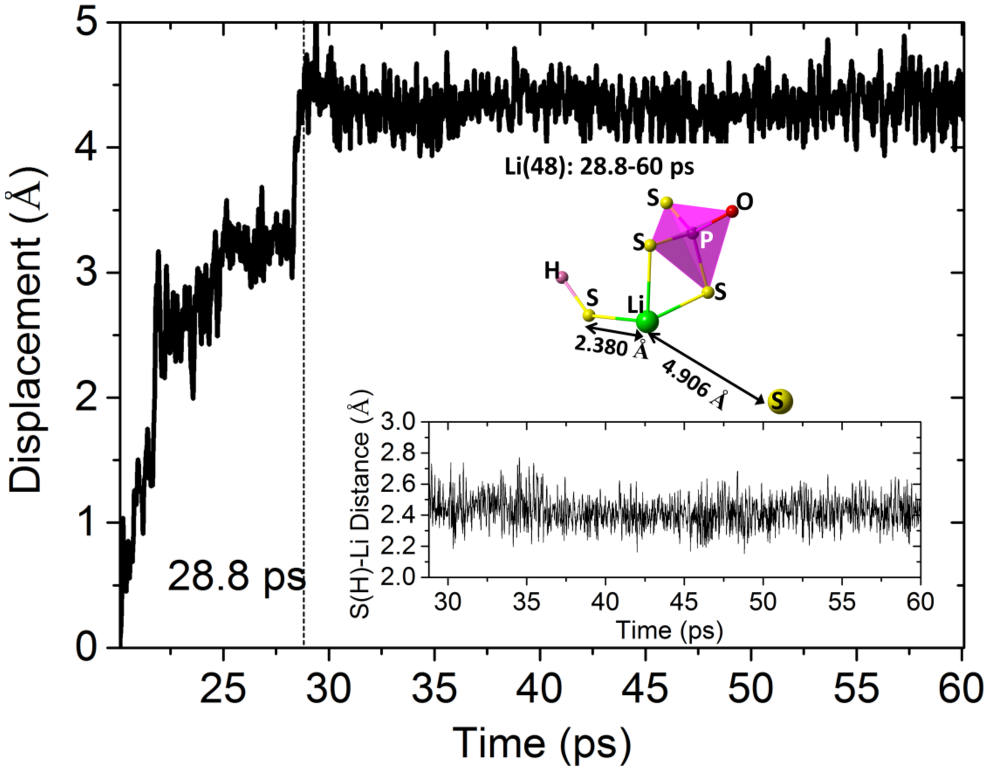

Supplementary Figure 8 Li(48) in the simulation cell of $Li_6POS_4(SH)$ trapped with the polyanion clusters at 28.8 ps, after escaping from its original sulfur block at the end of a long-distance diffusion event, as discussed in the paper. Li(48) then stay put in the trapping without participating any further diffusion event up to at least 60 ps, as shown by the calculated displacement and its distance with the interacting SH-cluster (inset).

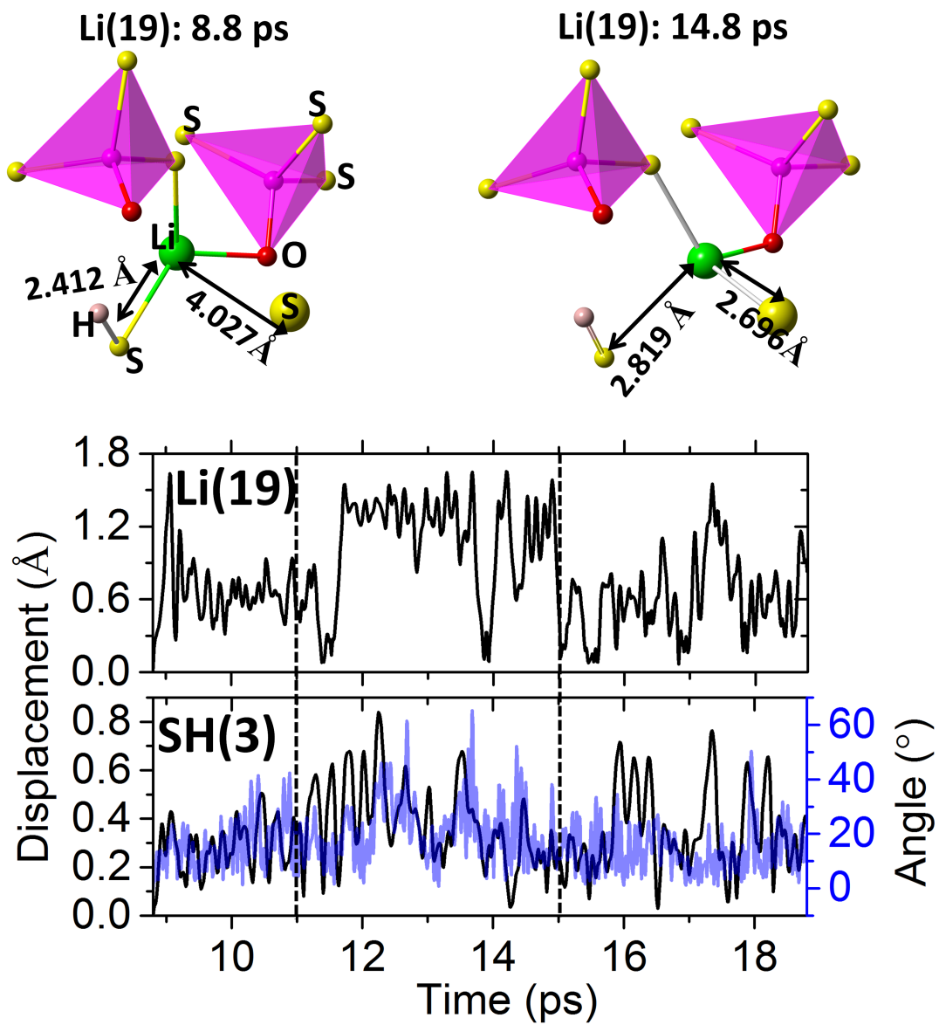

Supplementary Figure 9 Li(19) in the simulation cell of $Li_6POS_4(SH)$ trapped with the polyanion clusters at 8.8 ps without belonging to any of the sulfur blocks in the system. Later at 14.8 ps, it relocates to an adjacent sulfur block and participates in the follow-up diffusion events, as discussed in the paper. The process is due to the direct thermal excitation of Li(19) coupled with some rotational and translational dynamics of its interacted SH-cluster, as shown by the calculated displacement of Li(19) in upper panel and the rotational dynamics (angular rotation of the S-H vector in blue and translational displacement in black).

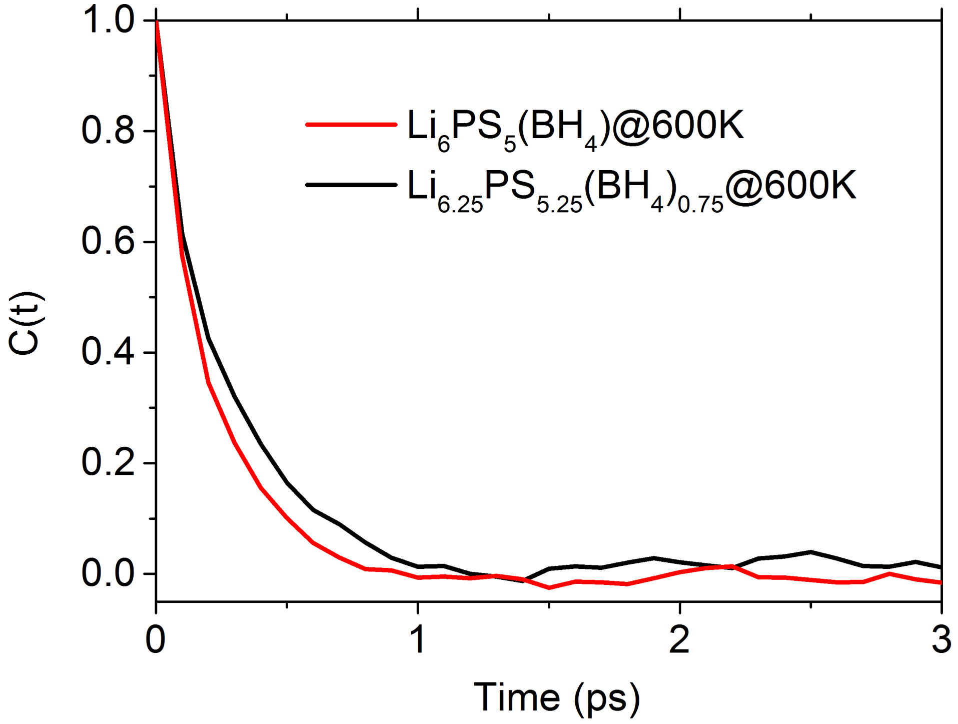

Supplementary Figure 10 Calculated angular time-correlation functions $C(t) = \langle \boldsymbol{u}(t) \bullet \boldsymbol{u}(t + t') \rangle$ for BH_4^- in $Li_6PS_5(BH_4)$ and $Li_{6.25}PS_{5.25}(BH_4)_{0.75}$, respective. $\boldsymbol{u}(t)$ is the unit vector from the center of mass of a cluster to its vertices at time *t*, *e.g.*, the unit vector from B to H in BH_4. $Li_6PS_5(BH_4)$ show significantly higher decorrelation rate than that of $Li_{6.25}PS_{5.25}(BH_4)_{0.75}$, suggesting its excessive cluster dynamics.

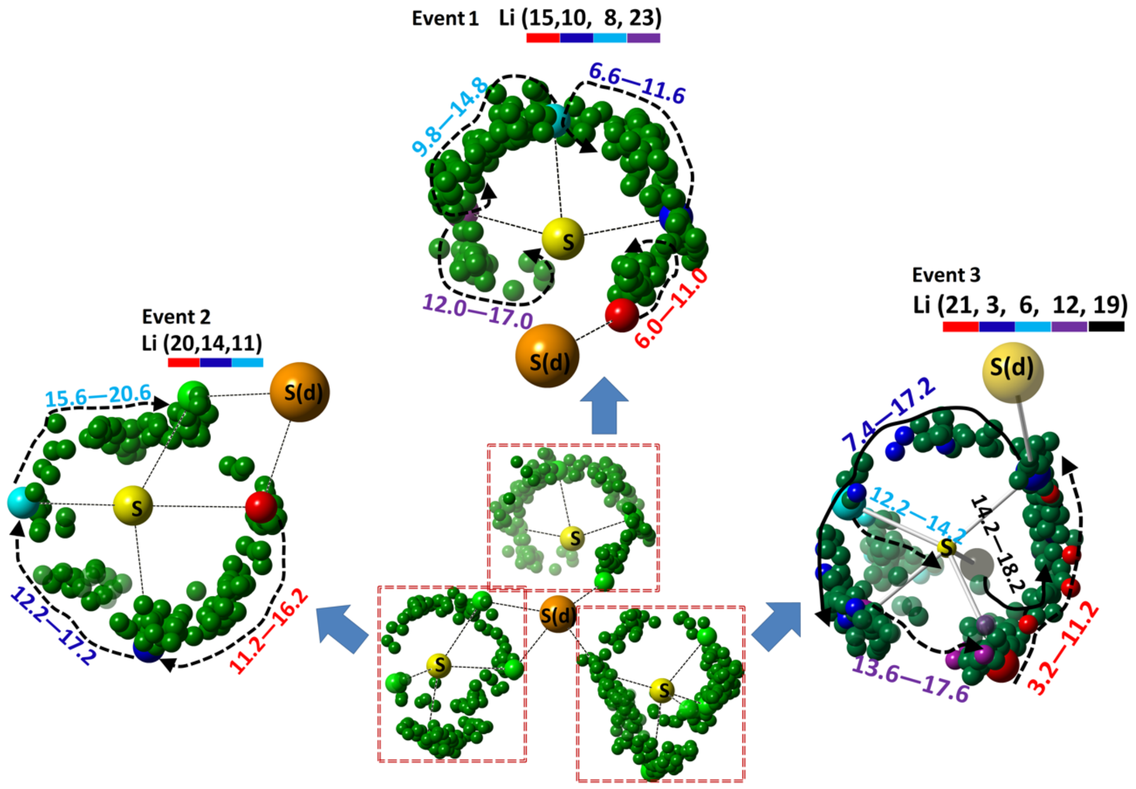

Supplementary Figure 11 Three combined events found for the nonstoichiometric lithium argyrodite $Li_{6.25}PS_{5.25}Cl_{0.75}$ at a simulation temperature of 500 K. S(d) represents the doped sulfur site in the structure. The Li-ion trajectories are in dark green. All three major events are found happening around the doped sulfur site. Event 1 involves the diffusion of Li(15) that originally bound to the doped sulfur to the nearby sulfur block (SLi_6), triggering a set of local diffusion in the block, according to the 'billiard-ball' mechanism as observed in the cluster-containing ALiSIC. Event 2 again shows a Li-ion Li(20), originally bound to the doped sulfur site, diffusing into a nearby sulfur block and triggering a set of intra-diffusions according to the billiard-ball mechanism. Event 3 is a typical 'revolving-door' mechanism featuring only local intra-diffusions in the same sulfur block.

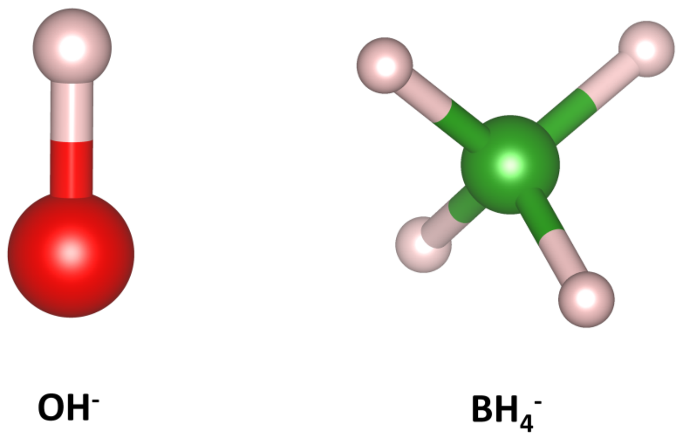

Supplementary Figure 12 Optimized gas-phase geometries of the cluster OH^- and BH_4^- which are initially introduced into first-principles structure search to find the ground-state structure of the studied lithium conductors. Oxygen is in red, hydrogen in pink and boron in green.

Supplementary Note 1: Statistical variance analysis of the low-temperature MD data.

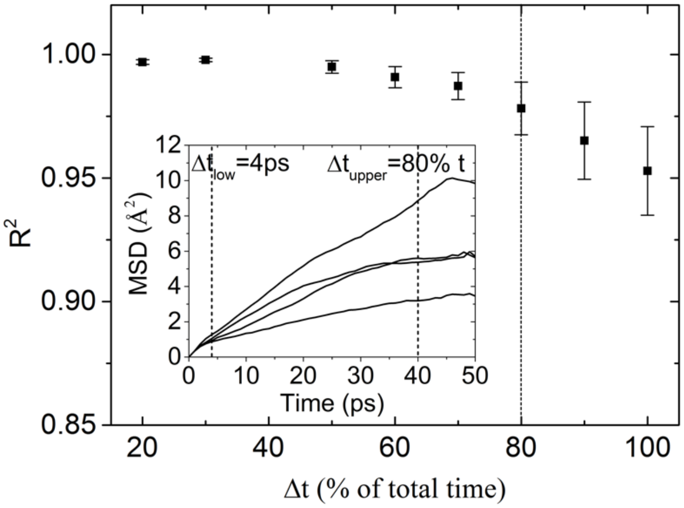

Supplementary Figure 13 Analysis to determine the lower and upper bounds of the linear fitting to the MSD data.

Following is the statistical variance analysis of the low-temperature MD data:

First, it is found [37] that the linear fit to the mean squared displacement (MSD) (Eq. 1) to obtain the ionic diffusivity (Eq. 2) should be performed on a time range $\Delta t_{low} \leq t \leq \Delta t_{upper}$ of the simulation. The data with $t < \Delta t_{low}$ are due to the harmonic vibration of Li-ions, and the data with $t > \Delta t_{upper}$ show large deviation due to the poor statistics at large Δt. Therefore, these data should be excluded from the linear fitting. As shown in the inset of Supplementary Figure 13, the value of Δt_{low} is determined to be 4 ps to exclude the so-called ballistic region of the data [37]. To determine Δt_{upper}, four MSD(t) curves (with each up to 50 ps, obtained by dividing a total MD simulation on $Li_{6.25}PS_{5.25}(BH_4)_{0.75}$ at 300 K of 250 ps into four well separated parts) are analyzed. As shown in Supplementary Figure 13, the goodness of the linear fit, R^2, with different upper fitting bound, of each MSD(t) curve is evaluated. It is shown that the value of R^2 begins to significantly deviate from 1 after $0.8 \times t_{tot}$. Therefore, Δt_{upper} is determined to be $0.8 \times t_{tot}$ in our analysis. Note that the MSD of Li-ions here is calculated from the MD data as,

$$MSD(\Delta t) = \frac{1}{N} TMSD(\Delta t), \quad (1)$$

where

$$TMSD(\Delta t) = \sum_{i=1}^{N} \frac{1}{N_{\Delta t}} \sum_{\Delta t=0}^{t_{tot}-\Delta t} |r_i(t' + \Delta t) - r_i(t')|^2$$

is the total mean squared displacement and *N* is the total number of Li-ions in the simulation cell. The diffusivity (*D*) is obtained from linear fitting to the MSD according to

$$D = \frac{MSD(\Delta t)}{2d\Delta t} D_{intercept} \quad (2)$$

with $d = 3$ the dimension of the conduction system.

Second, with the determined Δt_{low} and Δt_{upper}, we went on to evaluate the relative standard deviation (RSD) of the diffusivity, S_d/D_{true}, as defined in Ref. 37. S_d is the standard deviations of a set of fitted *D* values. These fits are shown in Supplementary Figure 14 for $Li_{6.25}PS_{5.25}(BH_4)_{0.75}$ at 300 K up to 100, 110, 120, 130, 140, 150, 180 and 200 ps, and for $Li_6POS_4(SH)$ at 400 K up to 80, 90, 100, 110 and 120 ps. D_{true} is calculated from the longest available MD (80% of the total time), which is 200 ps in the case of $Li_{6.25}PS_{5.25}(BH_4)_{0.75}$ at 300 K and 120 ps in the case of $Li_6POS_4(SH)$ at 400 K. The calculated RSD is found to be related to the total effective hops, N_{eff}, as in [37]

$$RSD = \frac{A}{\sqrt{N_{eff}}} + B \quad (3)$$

with A and B being the fitting parameters.

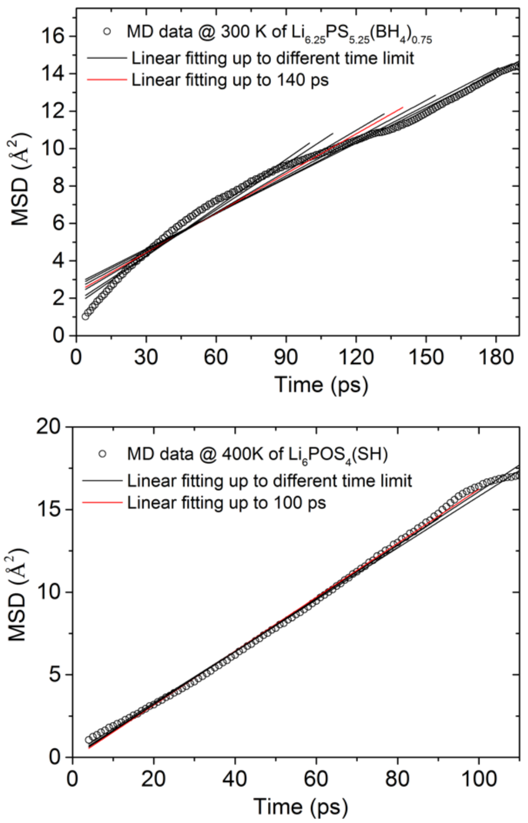

Supplementary Figure 14 Linear fitting to calculate the RSD of the diffusivity at different time limit or maximum TMSD (Eq. 1) or total effective hops N_{eff} (Eq. 3). The fittings used to obtain the ionic diffusivities at 300 and 400 K for $Li_{6.25}PS_{5.25}(BH_4)_{0.75}$ and $Li_6POS_4(SH)$, respectively, are marked in red, corresponding to 140 and 100 ps.

Here, N_{eff} can be calculated as $max[TMSD(\Delta t)]/a^2$ [37] with $max[TMSD(\Delta t)]$ the maximum of TMSD (Eq. 1) over the entire range and a = 2.0 and 1.8 Å for $Li_{6.25}PS_{5.25}(BH_4)_{0.75}$ and $Li_6POS_4(SH)$, respectively, equal to their corresponding threshold diffusion distances in the paper. Supplementary Figure 15 shows the calculated RSD *vs.* the N_{eff} and fitted by Eq. 3. It is found that the RSD of our calculated diffusivity of $Li_{6.25}PS_{5.25}(BH_4)_{0.75}$ at 300 K is 0.235 (linear fit up to 140 ps). The RSD of our calculated diffusivity of $Li_6POS_4(SH)$ at 400 K is 0.051 (linear fit up to 100 ps). These values are well below the convergence criteria of 0.3 proposed in Ref. 37-38.

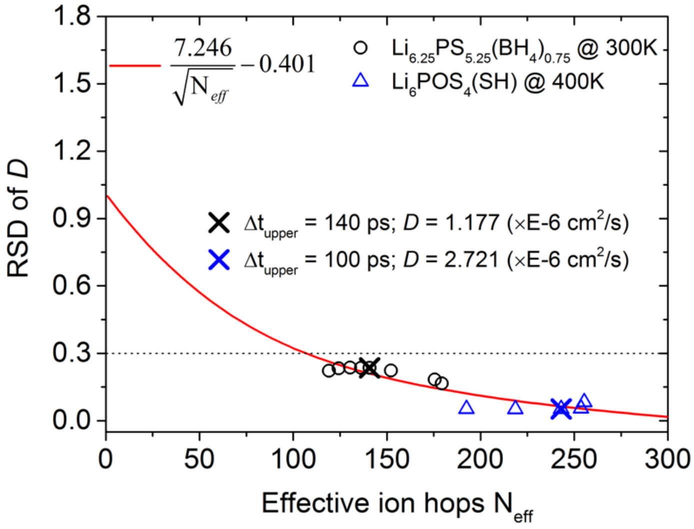

Supplementary Figure 15 Calculated relative standard deviation (RSD) of diffusivity *vs*. the total effective hops N_{eff}, fitted by Eq. 3. The highlight points by the cross correspond to the calculated diffusivities of $Li_{6.25}PS_{5.25}(BH_4)_{0.75}$ at 300 K and $Li_6POS_4(SH)$ at 400 K.

Supplementary Note 2: Estimation of the rotational barriers of BH_4^- and SH^- in the argyrodite-type structures.

The rotational barriers of BH_4^- and SH^- in their structures are estimated using nudged elastic band (NEB) method and selective dynamics. We use selective dynamics to fix the cluster under study to its designated angle, while allowing the other atoms in the supercell as well as the lattice parameters to fully relax during the NEB calculations. It is found that this method can characterize the expected rotation of the cluster of interest without involving rotations of the other clusters in the supercell during the relaxation. On the other hand, the atoms in the supercell and the lattice parameters can still be relaxed during the calculation. This is shown in Supplementary Videos 1-3 made from the trajectories from the NEB calculations. For the case of SH^-, one such cluster in the supercell is rotated about its C_2 axis (viewed as a rigid rod) up to 180 degrees with a 30-degree step size. Within each step, four intermediate states are calculated by the NEB method. A total of 24 intermediate states are calculated between 0 – 180 degrees. The results are shown in Supplementary Figure 16(A). The averaged rotational energy barrier per atom (two atoms are moving about the C_2 axis in the rotation) is 29 meV. For the case of BH_4^-, one such

cluster in the supercell is rotated about one of its C_2 axes (as a rigid tetrahedron) up to 90 degrees; and is rotated about one of its C_3 axes up to 120 degrees in another study. A total of 12 and 16 intermediate states are calculated in the C_2 and C_3 rotation, respectively. The results are shown in Supplementary Figure 16(B). It is found that the averaged rotational energy barrier per atom is 11 meV for C_2 rotation (with 4 H atoms moving) and 12 meV for C_3 rotation (with 3 H atoms moving).

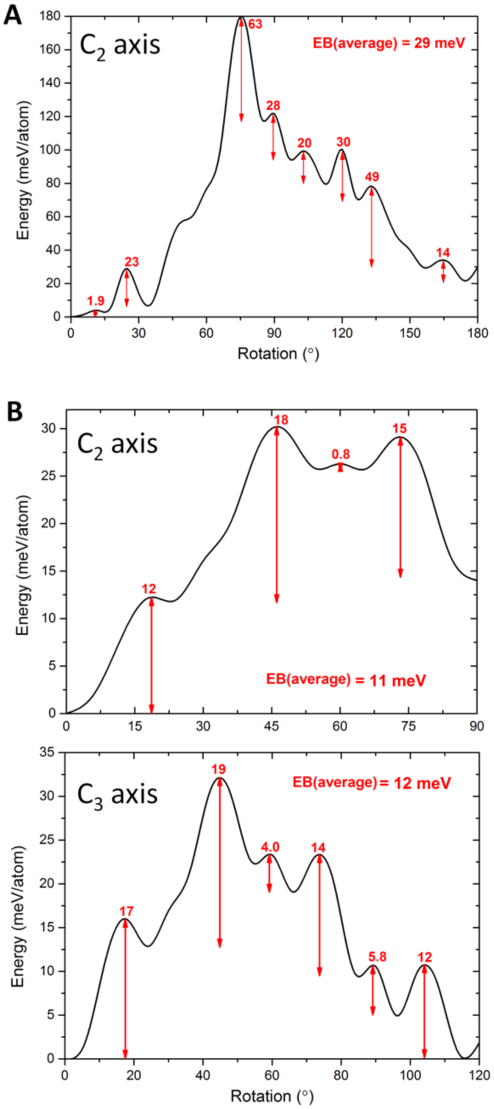

Supplementary Figure 16 Calculated energy profiles of rotation of the clusters. (**A**) Energy barriers (EB) of SH^- rotating as a rigid rod about its C_2 axis. (**B**) Energy barriers (EB) of BH_4^- as a rigid tetrahedron about its C_2 and C_3 axes, respectively. The small

difference in the starting and ending states are due to the relaxation of the atoms and lattice parameters of the supercell.

Supplementary Note 3: Description for the major Events in Figures 4-5 in the paper.

Event 1 involves only two Li-ions, including Li(2) and Li(41), and is characterized by intra-diffusions in the same sulfur-block. Li(2) begins an intra-diffusion at 46.8 ps and Li(41) begins its intra-diffusion towards the Li(2) position later at 47.2 ps. As shown in Fig. 4A, in the time period from 46.8 to 48.0 ps before the diffusion happens, Li(2) quickly switches its proximity towards SH(1) and SH(2) (as indicated by the SH inset in Fig. 4A). As shown in Fig. 4B, before the displacement of Li(2) (46.8--48.0 ps), SH(2) shows particularly weak rotational dynamics, while SH(1) which is also interacting with Li(2) shows some pronounced rotations. For the 'historical' intra-diffusion of Li(2) starting at 29.6 ps, as shown in Fig. 4C, its only related SH(2) cluster exhibits some level of rotational and translational dynamics throughout this time, and the dynamics actually become weaker during the next intra-diffusion of Li(2) (at 46.8-56.8 ps in Fig. 4A). On the other hand, correlation between the Li-ion transport and the rotational dynamics of the related cluster is still present. Li(41) is close to only the SH(1) cluster at the beginning and there is some pronounced rotational dynamics of SH(1) during the displacement of Li(41). Also, the pronounced 'dips' showing in the dynamics profile of SH(2) concur with those (around 50 and 54 ps) in the displacements of Li(2) and Li(41). There are both an 'active' element and a 'responsive' element in the dynamics of the SH cluster. The SH cluster shows active (rotational and translational) dynamics due to thermal excitation. Meanwhile, the cluster dynamics will also be responsive to the passing Li-ion. For example, as shown in Fig. 4B, there are some large rotations of SH(2) since 49 ps following Li(2)'s displacement.

Event 2 involves six Li-ions, including Li(48), Li(37), Li(32), Li(10), Li(11) and Li(19), and is characterized by cross-block diffusions involving three sulfur-blocks. Li(48) belonging to the first sulfur-block migrates a short distance at 8.8 ps. Along its trajectory, it pushes Li(37) to a short-distance intra-diffusion at 10.5 ps. From 20.1 to 28.2 ps, Li(48) undergoes a long-distance inter-diffusion out of the first sulfur-block, which is partially pushed by Li(11) which starts an intra-diffusion at 22.8 ps. As Li(48) moving towards the

second sulfur block, it triggers an intra-diffusion of Li(32) in that block at 20.5 ps. Li(32) then moves towards the Li(10) site in the same block, as Li(10) migrates away starting at 17.2 ps. The long distance diffusion of Li(48) ends around 30.1 ps and it stays out of any sulfur blocks up to at least 60.0 ps by interacting strongly with one SH cluster with the Li(48)-S(H) distance maintained throughout the time (as shown in Fig. S8 of SI). On the other hand, Li(19) which does not belong to any sulfur block at 8.8 ps is released by a SH cluster to its near-by sulfur-block (as shown in Fig. S9 of SI), and then begins an intra-diffusion inside the sulfur-block at 14.8 ps. These two cases of Li(48) and Li(19) show a 'docking/undocking' mechanism of the SH cluster acting on the Li-ion. From 59.2 to 63.0 ps, Li(19) undergoes another intra-diffusion towards Li(11) in the same block and pushes the latter to an intra-diffusion. Fig. 4D-F show the calculated displacements of Li(48), Li(11) and Li(19) together with the dynamics of their related SH clusters. The rotational dynamics of the SH clusters are found to be responsive to the Li-ion transport, rather than preceding the large Li-ion displacements. Relatively strong rotational dynamics of the SH clusters are only observed following the displacement of the Li-ion.

Event 3 involves another six Li-ions, including Li(27), Li(17), Li(5), Li(46), Li(13) and Li(39), and is characterized by cross-block diffusions involving three sulfur-blocks. Li(27) starts an intra-diffusion within the first sulfur-block at 16.0 ps. It pushes Li(17) in the same block and recoils. This triggers Li(17) to undergo a long-distance inter-diffusion starting after 16.2 ps. As it approaches the second sulfur block, it pushes Li(5) in the block, recoils and eventually stay in the block. Li(5) then starts an intra-diffusion at 22.6 ps and pushes Li(46) belonging to the same block to have an intra-diffusion from 22.8 to 28.8 ps. Starting at 30.2 ps, Li(13) of the third sulfur-block starts a long-distance inter-diffusion. As it relocates in the second block, it pushes Li(39) to begin an intra-diffusion in the block. Thus, an intra-diffusion can either promote a long-distance inter-diffusion by Li-Li repulsion when one moves close to another, or it can participate in a long-rang cross-block diffusion by forming a Li-Li repulsion 'chain' as described above. Again, as shown in Fig. 4G-I, the rotational dynamics of the SH clusters are found to be responsive to the Li-ion transport, rather than preceding any large Li-ion displacements, i.e., pronounced rotational dynamics are only observed following the Li-ion displacement.

For the case of $Li_{6.25}PS_{5.25}(BH_4)_{0.75}$, as shown in Fig. 5, **Event 4** involves four Li-ions, including Li(7), Li(43), Li(39) and Li(27), and is characterized by intra-diffusions in the same sulfur-block and near a doped sulfur (S2). Li(7) migrates a short-distance (< 2.0 Å) at 13.0 ps, followed by a large-displacement intra-diffusion pushed by Li(39) which starts to move after 22.2 ps. Li(39) begins a large-displacement intra-diffusion itself at 23.6 ps following Li(7) and ends up near the doped sulfur S(2). During the same period, Li(43) migrates towards the starting position of Li(39), while Li(7) moves towards Li(43) and recoils. Along its long-distance trajectory, Li(7) pushes Li(27) to an intra-diffusion starting at 29.4 ps. Here, we again see the behavior of one diffusion leading to anther by the Li-Li repulsion interaction. As shown in Fig. 5A, the short-displacement diffusion of Li(7) starting at 13.0 ps concurs with some rotations of BH_4(1). When Li(7) completes its large-displacement diffusion after 22.4 ps, it becomes closer to BH_4(6) and the cluster shows some strong rotational dynamics. Great thermally-excited rotational dynamics of the clusters are observed throughout the time.

Event 5 involves another four Li-ions, including Li(49), Li(32), Li(36) and Li(14), and is characterized by intra-diffusions inside the same sulfur-block and near a doped sulfur (S1). Li(49) undergoes a large-displacement intra-diffusion towards Li(32) at 43.0 ps. It pushes Li(32) to start an intra-diffusion at 46.0 ps and recoils. As Li(32) moving towards Li(36), it pushes the latter and recoils. Li(36) then begins an intra-diffusion at 47.4 ps, pushes Li(14) and recoils. Li(14) then begins an intra-diffusion at 48.6 ps towards the original site of Li(49). This behavior, which can be called a 'revolving door' mechanism, is characterized by a set of intra-diffusions with a time sequence that can passing the kinetic energy from one Li-ion to another. As we have seen before, a 'Li-Li repulsion chain' is again at play. Given that the four Li-ions in this event are moving consecutively when interacting with two clusters most of the time, it is difficult to clearly see the correlation between the Li-ions and the dynamics of the clusters, as shown in Fig. 5B. Especially, such correlation seems to be constantly interrupted by thermally-excited (rotational and translational) dynamics of the cluster.

Event 6 involves three Li-ions, including Li(4), Li(16) and Li(46), and is characterized by cross-block diffusions involving two sulfur-blocks and one doped sulfur (S1). Li(4) in the first sulfur-block begins a large-displacement inter-diffusion at 18.6 ps.

As it moving around the doped sulfur (S1) to the second sulfur-block, it pushes Li(16) and recoils back to dock at the doped sulfur (S1). Li(16) then starts an intra-diffusion at 23.6 ps towards Li(46). It pushes Li(46) to an intra-diffusion starting at 24.8 ps and recoils. Here, one inter-diffusion leads to a set of intra-diffusions across the sulfur-blocks, with once again the 'Li-Li repulsion chain' at play. In this event, the large-displacement diffusion of Li(4) is mediated by the doped sulfur (S1) instead of any BH_4 cluster. As shown in Fig. 5C, the translational dynamics of S1 is responsive to the motion of Li(4), becoming more pronounced after 18.6 ps when S1 starts to interact with Li(4) during the long-range diffusion.

Event 7 involves another six Li-ions, including Li(24), Li(28), Li(10), Li(40), Li(27) and Li(15), and is characterized by cross-block diffusions involving three sulfur-blocks and one doped sulfur (S2). Starting at 3.0 ps, Li(24) undergoes an intra-diffusion which pushes Li(28) to begin an intra-diffusion at 3.2 ps. Along its trajectory, it pushes Li(40) which starts an intra-diffusion and pushes Li(10) to begin a diffusion at 5.0 ps out of the sulfur block and ends up docking at the doped sulfur (S2). Li(28) proceeds with an inter-diffusion around the doped S(2) into the second sulfur block at 42.0 ps (as indicated in its calculated displacement plot in Fig. 5D). Along its trajectory, it pushes Li(27) in the third sulfur-block to an intra-diffusion at 43.0 ps and it also pushes Li(15) in the second sulfur-block to an intra-diffusion at 39.0 ps. Li(28) recoils and eventually resides in the second sulfur-block. Here, as has been observed in the previous events, an intra-diffusion leads to a long-distance inter-diffusion as well as a set of intra-diffusions across the sulfur-blocks according to a 'Li-Li repulsion chain'. As shown by Fig. 5D, there are some pronounced rotational dynamics of BH_4(6) concurring with the displacement of Li(28) between 3.2--5.0 ps. BH_4(4) shows strong rotations following the displacement of Li(28) when the two come near each other. These reflect the correlation between the Li-ion motion and the rotational dynamics of the cluster. After 42.0 ps, Li(28) is only interacting with the doped sulfur (S2), while both BH_4(4) and BH_4(6) still show strong rotational dynamics due to thermal excitation.
