# Supplementary material for: Argyrodite-type advanced lithium conductors and transport mechanisms beyond paddle-wheel effect
Source: Nat Commun. 2022 Apr 19;13:2078. doi: 10.1038/s41467-022-29769-5 (PMC9019101; doi:10.1038/s41467-022-29769-5)
Supplement: Supplementary file 3 — Description of Additional Supplementary Files [file 41467_2022_29769_MOESM3_ESM.pdf]

**Description of Additional Supplementary Files**

File Name: Supplementary Video 1
Description: Trajectory of the calculated rotation of OH (highlighted in red) about the $C_2$ axis. Li in green, S in yellow, P in violet, O in magenta and H in pink.

File Name: Supplementary Video 2
Description: Trajectory of the calculated rotation of $BH_4$ (highlighted in red) about the $C_2$ axis. Li in green, S in yellow, P in violet, B in light blue and H in pink.

File Name: Supplementary Video 3
Description: Trajectory of the calculated rotation of $BH_4$ (highlighted in red) about the $C_3$ axis. Li in green, S in yellow, P in violet, B in light blue and H in pink.
